# Reliability of self-reported health risk factors and chronic conditions questions collected using the telephone in South Australia, Australia

**DOI:** 10.1186/1471-2288-12-108

**Published:** 2012-07-26

**Authors:** Eleonora Dal Grande, Simon Fullerton, Anne W Taylor

**Affiliations:** 1 Population Research and Outcome Studies, Department of Medicine, University of Adelaide, Level 3, 122 Frome St, Adelaide, South Australia 5000, Australia

**Keywords:** Reliability, Telephone survey, CATI, Health risk Factors, Chronic conditions

## Abstract

**Background:**

Accurate monitoring of health conditions and behaviours, and health service usage in the population, using an effective and economical method is important for planning and evaluation. This study examines the reliability of questions asked in a telephone survey by conducting a test/retest analysis of a range of questions covering demographic variables, health risk factors and self-reported chronic conditions among people aged 16 years and over.

**Methods:**

A Computer Assisted Telephone Interviewing (CATI) survey on health issues of South Australians was re-administered to a random sub-sample of 154 respondents between 13-35 days (mean 17) after the original survey. Reliability between questions was assessed using Cohen’s kappa and intraclass correlation coefficients.

**Results:**

Demographic questions (age, gender, number of adults and children in the household, country of birth) showed extremely high reliability (0.97 to 1.00). Health service use (ICC = 0.90 95% CI 0.86-0.93) and overall health status (Kappa = 0.60 95% CI 0.46-0.75) displayed moderate agreement. Questions relating to self-reported risk factors such as smoking (Kappa = 0.81 95% CI 0.72-0.89) and alcohol drinking (ICC 0.75 = 95% CI 0.63-0.83) behaviour showed good to excellent agreement, while questions relating to self-reported risk factors such as time spent walking for physical activity (ICC 0.47 = 95% CI 0.27-0.61), fruit (Kappa_w_ = 0.60 95% CI 0.45-0.76) and vegetable consumption (Kappa_w_ = 0.50 95% CI 0.32-0.69) showed only moderate agreement. Self-reported chronic conditions displayed substantial to almost perfect agreement (0.72 to 1.00) with the exception of moderate agreement for heart disease (Kappa = 0.82 95% CI 0.57-0.99).

**Conclusion:**

These results show the questions assessed to be reliable in South Australia for estimating health conditions and monitoring health related behaviours using a CATI survey.

## Background

Telephone interviews are an effective and economical way to monitor health behaviours in the population. Data used in the planning and monitoring of health services and disease prevalence in populations should be as accurate as possible and assessing the reliability of questions is one way that accuracy or precision of the questions can be assessed and bias minimised. The aim of reliability testing is to make sure the responses to questions provide similar results and this is especially important if the questions are used in an on-going monitoring or surveillance system.

Papers have addressed the reliability of questions in telephone health survey questionnaires conducted by the Behavioral Risk Factor Surveillance System (BRFSS) in the United States [1-6]. The reliability tests of the BRFSS questionnaires have addressed a range of demographic variables and health risk factors, as well as specific issues such as ethnic monitories and women's health [6]. Demographic variables were found to have the highest reproducibility along with self-reported health, with health risk factors and ‘poor’ health days slightly less reliable although still at an acceptable level [1-5]. Knowledge or attitudinal variables were found to have lower reliability [2].

There remains a paucity of published reliability studies of Australian telephone health survey questionnaires to date. We previously published a reliability study of a telephone survey of South Australians (SA) in 1997 [7], and reported comparable findings to the BRFSS. Demographic questions showed the highest reproducibility, questions regarding health risk factors, such as smoking and alcohol consumption, showed substantial to almost perfect agreement, while chronic conditions variables were substantially reproducible where prevalence estimates were not close to zero. In addition, Brown et al [8] assessed reliability and validity of the Active Australia physical activity questionnaire and other physical activity measures and reported moderate to high levels of agreement. Nutrition questions administrated by self-completion have also been tested with the General Nutrition Knowledge Questionnaire having high test-re-test reliability [9]. Other relevant Australian studies have focused on children [10-13], older people [14,15], and on specific patient types (eg cataract) [16].

The South Australian Monitoring and Surveillance System (SAMSS) [17], is a chronic disease and risk factor surveillance system operated by the South Australian Department of Health. The aim of this study is to compare the reliability of questions asked in the SAMSS by conducting a test/retest analysis of a range of questions covering demographic variables, health risk factors and chronic conditions variables among people aged 16 years and over.

## Methods

### Study design

SAMSS is designed to systematically monitor the trends of diseases, health related problems, risk factors and other health services issues for all ages over time for the SA health system. This is a telephone monitoring system using the Computer Assisted Telephone Interview (CATI) method to detect emerging trends, to assist in the planning and evaluation of health policies and programs, and to assess progress in primary prevention activities. Data collected includes demographics, health risk and protective factors and chronic conditions.

Interviews are conducted on a minimum of 600 randomly selected people (of all ages) each month. All households in SA with a telephone connected and the telephone number listed in the Electronic White Pages (EWP) are eligible for selection in the sample. A letter introducing the survey is sent to the selected household and the person with the last birthday is chosen for interview. There are no replacements for non-respondents. Up to ten call backs are made to the household to interview the selected persons. Interviews are conducted by trained health interviewers.

Data are weighted by area (metropolitan/rural), age, gender and probability of selection in the household to the most recent SA population data so that the results are representative of the SA population. For participants aged less than 16 years, data are collected from an adult in the household, who has been nominated by a household member as the most appropriate person to answer questions on the child’s behalf.

### Test/retest study design

For this test/retest study, respondents were eligible to participate if they answered yes to being asked if they could be re-contacted to obtain further information regarding a health issue or in the event of a serious public health problem (n = 567) and aged 16 years and over (n = 551). The retest was conducted on a random sample of the eligible participants from 2009 November’s SAMSS survey (n = 495). Based on the literature [3,7,18] and costs, at least a third of the eligible sample would be approached for re-interview to obtain a sample size of at least 150 interviews. Call-back interviews started 14 days after the first interview with a mean 16.8 days, range 13 to 35 days. The same intensity that was used to contact participants for the first interview was used to contact the second time.

For the test/retest study, a selection of questions was asked of respondents (Table 1). Demographic variables included age, sex, country of birth [19], number of people living in the household aged 16 years and over, and aged 15 years or less. Health-related variables included current health status (SF1), risk and protective behaviours (body mass index [20] derived from height and weight, smoking status, alcohol consumption [21], nutrition intake, walking), and self-reported conditions (ever had high blood pressure or cholesterol, cardiovascular disease, asthma, osteoporosis, arthritis or diabetes).

**Table 1:** Socio-demographic, risk factor and self-reported chronic conditions questions

**Questions**	**Response categories**
Including yourself how many people aged 16 years and over live in this household?	
How many children (including babies) under 16 years live in your household?	
In which country [were you / was child’s name] born?	
In general, would you say [your] health is:	
Excellent ; Very good; Good; Fair; Poor	
In the last 12 months, how many times have you used a general practitioner in South Australia?	
Have you ever been told by a doctor that you have diabetes?	
Have you ever been told by a doctor that you have asthma?	
Have you ever been told by a doctor that you have had any of the following conditions?	Heart attack; Angina; Heart disease; Stroke; None of the above
Have you ever been told by a doctor that you have arthritis?	Yes, Osteoarthritis; Yes, Rheumatoid Arthritis; Yes, Juvenile Rheumatoid Arthritis (JRA); Yes, other (specify); No, don’t have arthritis; Yes, don’t know type
Have you ever been told by a doctor that you have osteoporosis?	
Have you ever been told by a doctor or a nurse that you have high blood pressure?	
Have you ever been told by a doctor or a nurse that you have high cholesterol?	
What is your height without shoes? (Centimetres or Feet : Inches)	
What is your weight? (Undressed in the morning)? (Kilograms (Kg) or Stones : Pounds)	
In the last week, how many times have you walked continuously, for at least 10 minutes, for recreation, exercise or to get to or from places?	
What do you estimate was the total time that you spent walking in this way in the last week?	
The following questions are about tobacco smoking. This includes cigarettes, cigars and pipes. Which of the following best describes your home situation?	My home is smoke free (includes smoking is allowed outside); People occasionally smoke in the house; People frequently smoke in the house
Which of the following best describes your smoking status?	I smoke daily; I smoke occasionally; I don’t smoke now but I used to; I’ve tried it a few times but never smoked regularly; I’ve never smoked
How often do you usually drink alcohol?	
A Standard Drink is equivalent to a schooner or midi of full strength beer, a glass of wine or a nip of spirits. On a day when you drink alcohol, how many drinks do you usually have?	
How many serves of vegetables [do you] usually eat each day? A ‘serve’ is ½ cup cooked vegetables or 1 cup of salad.	
How many serves of fruit [do you] usually eat each day? A ‘serve’ is 1 medium piece or 2 small pieces of fruit or 1 cup of diced pieces.	

The initial interviews took an average of 19 minutes (range 3 to 42 minutes) and each test/retest interviews took an average of 5 minutes (range 3 to 11 minutes).

### Data analyses

Response rates were calculated for both surveys. Those who participated in the test/retest study were compared to the initial sample of SAMSS participants aged 16 years and over. For each categorical variable, the observed agreement (percentage of respondents who agree with themselves) and expected agreement (percentage of respondent who agree with themselves which would be expected by chance) was calculated. Cohen's kappa statistic (κ) [22,23] was used to calculate reliability for categorical variables; weighted kappa (κ_w_) [24] was used for ordinal variables (eg. alcohol risk) with user-defined weighting system, and Intraclass Correlation Coefficients (ICC) was used for continuous variables (eg. age, BMI). 95% confidence intervals (CI) were calculated for each measure of agreement. Kappa is a measure of agreement beyond that is expected by chance, calculated by (observed agreement-chance agreement)/(1-chance agreement). Kappa statistics is affected by prevalence (very low or very high) and skewed data which can produce low values of kappa. It should be noted that the interpretation of agreement results on continuous variables using ICC should be performed with caution. ICC is a function of the range of the continuous variable being assessed. Larger range increases the ICC, independent of the actual differences between the measures being compared. Reliability values of less than 0.20 were considered ‘poor’ agreement, between 0.21-.40 as ‘fair’, between 0.41 and 0.60 as ‘moderate’, between 0.61 and 0.80 as ‘good’ agreement and between 0.81 and 1.00 as ‘excellent’ agreement [25]. Data from the CATI system were analysed in SPSS version 17.0 [26] and Stata (Version 9.0) [27].

Ethical approval for the project was obtained from University of Adelaide (ethics approval number H-182-2009). All participants gave informed consent.

## Results

Overall n = 626 respondents participated in SAMSS in November 2009 (response rate = 64.9%), with n = 495 (89.8%) of the people aged 16 years and over (n = 551) willing to be recontacted. Every third participant was selected to be recontacted (n = 165). In total 154 (93.3%) of those who were selected to be recontacted were reinterviewed.

Overall, 57.8% of participants of the retest study were female and the average age was 58 years (range 16 to 93). Table 2 displays the demographic differences between those who were reinterviewed (n = 154) and those who did not want to be recontacted, eligible but not selected, and eligible and selected but were not re-interivewed (n = 397).

**Table 2:** Demographic variables among the retest study participants and those who did not participated in retest study, 16 years and over, South Australia, Australia

	**Retest Participants**		**Did not participated in retest**^**a**^		**P value**
	**n**	**%**	**n**	**%**	
**Sex**					0.64
Male	65	42.2	159	40.1	
Female	89	57.8	238	60.0	
**Age group**					0.25
16 to 34 years	16	10.4	56	14.1	
35 to 54 years	33	21.4	100	25.2	
55 + years	105	68.2	241	60.7	
**Household income**					0.34
Up to $20,000	40	26.0	74	18.6	
$20,001 to $40,000	30	19.5	79	19.9	
$40,001 to $60,000	15	9.7	57	14.4	
$60,001 to $80,000	17	11.0	39	9.8	
$80,001 or more	32	20.8	83	20.9	
Not stated	20	13.0	65	16.4	
**Employment status**					0.60
Full time employed	48	31.2	101	25.4	
Part time employed	25	16.2	71	17.9	
Unemployed	4	2.6	10	2.5	
Economically inactive	77	50.0	215	54.2	
**Highest education level**					0.36
Degree or higher	29	18.8	81	20.4	
Trade/certificate/diploma	43	27.9	88	22.2	
No schooling to secondary	82	53.3	228	57.4	
**Area of Residence**					0.67
Metropolitan Adelaide	100	64.9	267	67.2	
SA Country	54	35.1	130	32.8	
	154	100.0	397	100.0	

NS = P > 0.005 using Chi Square.

a: Non-retest participants (16+ years) included participants who did not wish to be recontacted at initial interview, eligible participants but not selected for retest study, and eligible participants who were selected but did not complete retest inteview.

Table 3 presents estimates of reliability for all assessed variables. In general, reproducibility to demographic characteristics was extremely high especially for age (ICC 1.00); gender (κ 1.00); country of birth (κ 0.98); number of people in household aged 16 + (ICC 0.97) and number of people in household aged less than 16 years (ICC 0.99). Health service use, displayed good agreement with a correlation coefficient of 0.90. Overall health status displayed a moderate agreement beyond chance (κ_w_ 0.60) but the observed agreement was 85.8%, which indicated that the kappa statistic was affected by the low proportion reporting ‘poor’ health.

**Table 3:** Reliability estimates on demographic, health risk factors and co-morbidity of people aged 16 years and over in South Australia, Australia

	**Response at 1**^**st**^**interview (%)**	**Response at 2**^**nd**^**interview (%)**	**Observed Agreement (%)**	**Expected Agreement (%)**		**Reliability value**
**Age**					ICC	1.00
16-34 years	10.4	10.4				
35-54 years	21.4	21.4				
55 years and over	68.2	68.2				
**Gender**			100.0	51.2	Kappa	100.0
Male	42.2	42.2				
Female	57.8	57.8				
**Number of people in household aged 16 +**					ICC	0.97 (0.97 - 0.98)
**Number of people in household aged < 16**					ICC	0.99 (0.99 -1.00)
**Region of birth**			99.4	62.1	Kappa	0.98 (0.95 – 0.99)
Oceania and Antarctica	76.6	77.3				
North West Europe	16.9	16.2				
Southern and Eastern Europe	2.6	2.6				
North East Asia	0.6	0.6				
Southern and Central Asia	1.9	1.9				
Americas	1.3	1.3				
**Health service used in last 4 weeks**						
Mean (sd)	4.7 (4.917)	4.8 (5.937)			ICC	0.90 (0.86 - 0.93)
Range	0-30	0-52				
Number of times provided	95.5	99.4				
Not stated	4.5	0.6				
**HEALTH STATUS**						
**Overall health status**			85.8	64.3	Weighted Kappa^a^	0.60 (0.46 - 0.75)
Excellent	13.0	13.0				
Very good	36.4	39.6				
Good	31.8	34.4				
Fair	16.2	9.7				
Poor	2.6	3.2				
**Health conditions (ever been diagnosed by a doctor)**						
Asthma (ever)	14.3	13.0	93.5	76.4	Kappa	0.72 (0.56 - 0.87)
Diabetes	8.4	8.4	100.0	84.5	Kappa	1.00
Arthritis	26.0	27.3	92.2	60.9	Kappa	0.80 (0.693 - 0.91)
Heart attack	6.5	6.5	97.4	87.9	Kappa	0.79 (0.583 - 0.99)
Angina	3.2	4.5	98.7	92.5	Kappa	0.83 (0.592 - 0.99)
Heart disease	3.9	3.2	96.8	93.1	Kappa	0.52 (0.17 - 0.89)
Stroke	2.6	1.9	99.4	95.6	Kappa	0.85 (0.57 - 0.99)
Osteoporosis	7.1	5.8	99.4	87.9	Kappa	0.79 (0.58 - 0.99)
**HEALTH-RELATED RISK FACTORS**						
**Ever been told by doctor or nurse you have …**						
High blood pressure	35.1	37.0	91.6	53.9	Kappa	0.82 (0.72 - 0.91)
High cholesterol	38.3	37.7	90.3	52.9	Kappa	0.79 (0.69 - 0.89)
**Height (cms)**						
Mean (sd)	169.6 (9.735)	169.2 (9.429)			ICC	0.97 (0.96 - 0.98)
Range	147.3-193.0	147.3-193.0				
Height provided	98.7	98.7				
Not stated	1.3	1.9				
**Weight (kgs)**						
Mean (sd)	77.3 (17.117)	77.5 (17.150)			ICC	1.00 (0.99 - 1.00)
Range	44.5-151	44.5-150				
Weight provided	96.8	96.1				
Not stated	3.2	3.9				
**BMI (derived)**^**c**^						
Mean (sd)	26.7 (4.918)	27.1 (4.980)			ICC	0.98 (0.98 - 0.99)
Range	17.1-47.8	18.5-47.5				
BMI calculated	96.1	95.4				
Not stated	3.9	4.6				
**Body Mass Index (derived)**			97.9	70.2	Weighted Kappa^a^	0.93 (0.75 - 1.00)
Underweight (<18.5)	0.7	-				
Normal (> = 18.5 & < 25)	43.5	40.4				
Overweight (> = 25 & <30)	32.0	34.2				
Obese (> = 30)	23.8	25.3				
**Which best describes your home smoking situation….**			96.1	86.0	Kappa	0.59 (0.33 - 0.85)
My home is smoke free (includes smoking is allowed outside)	94.2	94.3				
People occasionally smoke in the house	3.9	3.8				
People frequently smoke in the house	1.9	1.9				
**Which of the following describes your smoking status..**			84.4	38.2	Kappa	0.75 (0.66 - 0.84)
I smoke daily	8.4	7.8				
I smoke occasionally	2.6	3.9				
I don't smoke now but I used to	27.3	29.2				
I've tried it a few times but never smoked regularly	6.5	6.5				
I've never smoked	55.2	52.6				
**Smoking status (derived)**			89.0	42.4	Kappa	0.81 (0.72 - 0.89)
Smoker	55.2	52.6				
Ex -smoker	33.8	35.7				
Non -smoker	11.0	11.7				
**Alcohol consumption**						
**Number of days a week drink alcohol …**			83.0	37.5	Weighted Kappa^b^	0.75 (0.64 - 0.85)
Don't drink alcohol	20.1	25.3				
Less than once per week	26.0	18.8				
1	14.9	18.2				
2	11.7	9.1				
3	5.8	8.4				
4	3.2	3.2				
5	4.5	6.5				
6	0.6	1.3				
7	13.0	9.1				
**Number of standard drinks per day when drinking....**					ICC	0.75 (0.63 - 0.83)
Mean (sd)	2.5 (2.562)	2.3 (2.013)				
Range	1-20	1-12				
Number of drinks provided	100.0	100.0				
Not stated	-	-				
**Risk to health due to alcohol consumption (short term) (derived)**			95.0	85.3	Weighted Kappa^b^	0.66 (0.45 - 0.87
Non- drinker	20.1	25.3				
Low risk	54.5	51.3				
Risky	23.4	20.8				
High risk	1.9	2.6				
**Serves vegetables per day**					ICC	0.64 (0.50 - 0.74)
Mean (sd)	3.0 (1.453)	3.0 (1.600)				
Range	0.5 - 8.0	0 - 8.0				
Serves per day provided	100.0	98.7				
Not stated	-	1.3				
1 or less serves **(derived)**	14.3	17.1	93.2	86.2	Weighted Kappa^a^	0.50 (0.32 - 0.69)
2 to 4 serves	69.5	66.4				
5 or more serves	16.2	16.4				
**Serves fruit per day**					ICC	0.83 (0.77 - 0.88)
Mean (sd)	1.7 (1.034)	1.6 (1.069)				
Range	0 - 8.0	0 - 8.0				
Serves per day provided	100.0	100.0				
Not stated	-	-				
None to 1 serves **(derived)**	46.1	54.5	96.0	89.0	Weighted Kappa^a^	0.60 (0.45 - 0.76)
2 or more serves	53.9	45.5				
**Total time walking in a week (mins)**					ICC	0.47 (0.27 - 0.61)
Mean (sd)	120.4 (163.3)	99.3 (163.1)				
Range	0-1200	0-1260				
Minutes/hours per week provided	100.0	97.4				
Not stated	-	2.6				

Note : ICC Intraclass correlation coefficient.

a: weighting system used: 1 (perfect agreement), 0.8 (difference of 1 category) and 0 (difference of 2 to 4 categories).

b: weighting system used: 1 (perfect agreement), 0.9 (difference of 1 category) and 0 (difference of 2 to 8).

c: only calculated for respondents aged 18 years and over (n = 153).

Overall health conditions displayed excellent observed agreements (92% to 100%) and good to excellent agreement beyond chance. Data on diabetes showed the highest reliability with a kappa value of 1.00. Reliability was lowest for heart disease (κ 0.53) but had excellent observed agreement (96.8%) which would be due to the low prevalence of the condition (3.9% and 3.2%). Similarly other health conditions had kappa values (good to excellent agreement beyond chance) between 0.72 (asthma) and 0.80 (arthritis), and had excellent observed agreements. Again, the low prevalence of cardiovascular diseases (heart attack, angina and stroke) and osteoporosis affected the kappa statistic when the observed and expected agreements were excellent.

Overall health risk factors displayed fair to excellent agreement with the highest being a correlation coefficient value of 0.98 for BMI and lowest being a correlation coefficient of 0.47 for the total time (minutes) walking per week. Overall protective factors displayed fair to moderate agreement beyond chance in serves of vegetables per day (κ_w_ 0.50) and serves of fruit per day (κ_w_ 0.60).

## Discussion

SAMSS has contributed to the monitoring of departmental issues, key risk factors and population trends in priority chronic disease and related areas thereby guiding investments, identifying target groups, providing important program and policy information, and assessing outcomes. Potential bias from differing probabilities of selection in the sample is addressed by weighting by age, gender, probability of selection in the household, and area of residence to the most recent estimates of residential population, derived from census data by the Australian Bureau of Statistics. Additional bias may result if the questions are not reliable, that is the questionnaire will not always result in the same response when repeatedly administered to the same respondents. Undertaking this study to determine this source of bias determined that substantial to almost perfect reliability for the questions asked in SAMSS that were tested. These findings are consistent with published literature of BRFSS survey questions [1-6] and a previous reliability study of health survey questions asked in South Australia [7].

The high level of reliability for demographic variables reflects survey administration protocols which ensured that the respondent was in fact the original respondent, resulting in excellent agreement beyond chance on sex and age. The small variation in household composition would be expected in a random sample of the population over such a short time frame. This finding is consistent with our previous reliability study [7] and BRFSS study [3].

It is reasonable to expect some variation between surveys for behavioural related variables such as physical activity, smoking or alcohol consumption, with a change between surveys leading to a lower level of agreement for these variables. This retest was performed a minimum of 13 days following the initial survey (mean 16.8; SD 3.6). This length was long enough so that respondents were not expected to have remembered the answers that they gave to the first survey and also unlikely that answers would change significantly, making it possible that the two surveys could be considered independent. Despite this reasoning, real changes could have occurred between interviews, which would weaken the reliability estimates. Notwithstanding, any differences could be the result of social desirability and the subjective need to report knowledge rather than actual behaviour. This could explain the lower kappa scores for smoking status (κ 0.81) smoking situation at home (κ 0.59) and alcohol consumption (reliability values from 0.66 to 0.75). Our results for smoking status, which had an observed agreement of 89.0%, is similar to our previous study (κ 0.92) and other studies with kappa values of current smoking of 0.85 [3], 0.90 [4] and 0.83 [5]. Smoking situation at home had moderate agreement beyond chance (κ 0.59) but the observed agreement (96.1%) was excellent. This low kappa value would be due to the responses to the categories being close to 0% or 100% which affects the kappa statistic. The results for alcohol consumption had moderate agreement beyond chance which was similar to our previous finding [7]. Similar to smoking, the risk of harm to health due to alcohol consumption had a weighted kappa value of 0.66 but high observed agreement of 95.0%. This suggests that smoking status and alcohol consumption are relatively reliable due to the excellent observed agreement despite the moderate kappa values. The total time estimated walking had the lowest level of agreement (ICC 0.47) which was similar to another Australian study which had reliability score of 0.56 [28] and our previous study (κ=0.54 95% CI 0.37-0.70) and other BRFSS study [4] with kappa values of 0.54 for sedentary lifestyle and 0.56 for inactivity. The fair agreement could be due to a real change in total time walking between the two interviews or the ability to recall actual time on walking was not good. It should be noted that outliers can greatly influence the ICC producing low values and the possibility of converting the values to categories can result in a higher reliability values [29].

Similarly, while questions relating to self-reported risk factors such as fruit and vegetable consumption showed only fair to moderate agreement beyond chance this could be due to changes in behaviour following the first interview. However in both cases, the number of serves of both fruit and vegetables appeared to decrease following the first survey. It is also possible that participants modified their answers the second time due to providing social desirable or knowledge answers as a result of major advertising campaigns, Go for 2&5®, that was conducted in SA and other states to promote the recommended 2 serves of fruit and 5 serves of vegetables per day. Or participants do not have the ability to recall all of the fruit or vegetables consumed per day or to quantify fruit and vegetables into serve sizes.

Somewhat unexpected is the excellent reliability for height, weight and BMI (ICC ranging from 0.97 to 1.00), and the excellent observed agreement (97.9%) and agreement beyond chance in the derived BMI categories (κ_w_ 0.93). These results are consistent with our previous study which the weighted kappa value for the BMI categories had of 0.89 (95% CI 0.84-0.92) [7] and a BRFSS study which reported excellent reliability for height, weight and BMI (Pearson’s *r* 0.84 to 0.94). We have previously addressed the reliability of self-reported height and weight when compared to clinic measurements [30] but this study has shown that BMI, as a broad measurement of adiposity, is reliable when collecting self-reported data over the telephone.

The questions relating to chronic condition variables, and high blood pressure and cholesterol displayed excellent observed agreements and good to excellent agreement beyond chance with the exception of heart disease. As stated previously, the low prevalence of heart disease affects the kappa statistic when the observed and expected agreements were shown to be excellent. Hence the questions to obtain prevalence estimates on health conditions, and high blood pressure and cholesterol are reliable in telephone surveys. Health service use displayed excellent agreement and one would expect some variation in the time period of this study. Overall health status use had moderate agreement by chance (κ 0.60) but good observed agreement (85.8%). The kappa value is lower than a BRFSS study (κ 0.75). This low kappa value found in this study could be partly because the respondent’s health could have changed between the two time periods and the low proportion reporting ‘poor’ health which affects the kappa statistic.

Weaknesses of the study include the possible bias from a willingness to participate, although comparison with SAMSS November sample does not indicate this to be the case. In addition, the retest data were not weighted so any prevalence estimates should be used with the utmost caution. Although telephones are connected to a large number of Australian households, not all are listed in the EWP (mobile only households and silent numbers). In 2008 in South Australia, 9% of households are mobile only and 69% of households do not have their landline or mobile number listed in EWP [31]. Previous work undertaken in 1999 has shown that inclusion of unlisted landline numbers in the sampling did not impact on the health estimates [32]. However, mobile only households are increasing in South Australia and following international trends, and a very small proportion (7%) elect to have their mobile number listed in EWP [31]. Presently, the exclusion of this group from the current sampling frame may be small in relation to the health estimates obtained using EWP. However, the characteristics of people living in mobile-only households are distinctly different and the rising proportion in the number of mobile-only households is not uniform across all groups in the community. Given these sampling issues, there is potential bias in the results obtained in this study.

The response rate of nearly 65% is moderately acceptable for this type of survey but the potential for survey non-response bias is acknowledged. Response rates are declining in surveys based on all forms of interviewing [33] as people have become more active in protecting their privacy. The growth of telemarketing has disillusioned the community and diminished the success of legitimate social science research by means of telephone-based surveys.

## Conclusion

This study has shown that there is a high level of reliability associated with the 20 questions routinely asked in a regular SA risk factor and chronic disease surveillance system. Although high reliability levels were apparent it should be remembered that this does not equate to high validity. Further studies are required to establish the validity of the some of the questions asked in telephone surveys such as SAMSS. Nevertheless we conclude that these questions provide a reliable tool for assessing these indictors using the telephone as the data collection method.

## Abbreviations

SA, South Australia; SAMSS, South Australian Monitoring and Surveillance System; CATI, Computer Assisted Telephone Interviewing; EWP, Electronic White Pages; BMI, Body mass index; BRFSS, Behavioral Risk Factor Surveillance System; ICC, Intraclass Correlation Coefficients; SD, Standard deviation; CI, Confidence intervals; κ, Cohen's kappa statistic; SPSS, Statistical Package for Social Sciences.

## Competing interests

The authors declared that they have no competing interests.

## Authors’ contribution

EDG, Participated in the design and co-ordination of the study, performed statistical analyses, drafted the manuscript. SF, Participated in the design and co-ordination of the study and involved in the drafting of the manuscript. AWT, Participated in the design and co-ordination of the study, drafted the manuscript. All authors read and approved the final manuscript.

## Pre-publication history

The pre-publication history for this paper can be accessed here:

http://www.biomedcentral.com/1471-2288/12/108/prepub
